# Supplementary material for: Evaluation of Dynamic Changes of Volatile Organic Components for Fishmeal during Storage by HS-SPME-GC-MS with PLS-DA
Source: Foods. 2024 Apr 23;13(9):1290. doi: 10.3390/foods13091290 (PMC11083336; doi:10.3390/foods13091290)
Supplement: Supplementary file 1 [file foods-13-01290-s001.zip › foods-2941717-supplementary.pdf]

Table S1 The species and relative content of VOCs during storage

Domestic fishmeal																	
CAS	Compound	Relative content (μg/kg)															
	CID	0d	2d	4d	6d	8d	10d	12d	14d	16d	18d	20d	22d	24d	26d	28d	30d
91-20-3	931	15.28±4.82	27.05±0.26	9.99±1.84	12.39±4.48	11.88±4.10	9.81±1.04	13.74±2.31	13.74±1.65	15.67±5.04	13.54±1.16	13.50±2.39	12.90±3.79	16.18±3.24	9.36±0.49	11.88±5.37	39.11±22.43
18433-98-2	519564	4.00±1.35	2.00±0.40	3.61±0.74	2.63±0.77	2.40±0.39	2.80±0.64	2.10±0	2.56±0.28	2.91±0.66	2.38±0.05	3.14±0.26	1.73±0	5.06±0	2.67±0	2.87±0	4.02±0.85
91-53-2	3293	1.18±0.13		1.13±0.10	1.42±0.22	2.49±0.49	1.74±0.71	1.89±0.05	1.33±0.50	1.85±0.49	1.38±0.04	1.75±0.05	2.61±0	1.24±0.16	0.94±0.14	1.55±0.28	2.10±0
123-32-0	31252	3.00±0		0.88±0				2.04±0			1.38±0.60	1.83±0					1.33±0
1008-88-4	13886	1.60±0.90	3.13±0.11	2.18±0.39	2.38±0.52	3.02±0	3.02±0.62	3.26±0.63	2.90±0.44	3.42±0.43	3.58±0.14	3.04±0.82	4.59±1.73	5.03±0.72	3.27±0.11	3.69±1.28	8.55±3.92
55044-09-2	583842	4.19±1.27												5.19±0			
90-12-0	7002	3.16±1.47	1.91±0.25	1.65±0	2.26±0.86	2.68±1.17	1.77±0.23	3.96±0.62	3.62±1.15	3.78±0.80	2.84±0.29	3.41±0.04	2.38±0.71	4.63±1.72	2.34±0.29	3.27±1.51	8.44±2.91
13360-	25916	9.88±0					1.28±	4.56±0	2.35±0.	2.68±0		0.73±0	3.18±	2.52±0			

65-1							0	.39	87				0				
95-20-5	7224		1.58±0.04								2.51±0.09	2.35±0	1.42±0				5.14±0
13067-27-1	83101		2.31±0			2.18±0.65							2.50±0	2.42±0			
1746-11-8	101130		2.32±0														
1558-17-4	15257		1.92±0		2.97±0	1.29±0											2.14±0
2349-70-4	3083760		1.18±0														
4466-24-4	20534		1.30±0			1.19±0		1.91±0			1.96±0	1.90±0	0.85±0				
1072-83-9	14079			0.90±0							0.95±0		1.19±0				
2941-72-2	76254			1.08±0							1.51±0						
1000380-56-7	68109			3.41±0			1.66±0	6.22±0.63					1.93±0	0.97±0	8.05±1.27		
91-57-6	7055			1.06±0										1.21±0			
1000117-04-4	590619			1.00±0					1.06±0								

3208-16-0	18554				1.63±0	1.02±0	1.33±0	0.93±0		0.98±0	2.16±1.34	1.1±0		8.01±0		1.41±0	16.68±0
3777-69-3	19602				0.69±0				0.75±0		0.62±0						
59958-20-2	181250					3.01±0.65											
164984-44-5	188454					1.26±0.29	0.80±0.05										
1008-89-5	13887					2.51±0											
28712-62-1	120028								3.79±0								
1122-58-3	14284									1.25±0							
695-34-1	1533									1.02±0							
56599-50-9	543312																7.94±0
104-46-1	637563											1.35±0.10					
73285-35-5			5.67±0														15.12±0
629-78-7	12398	64.40±10.57	96.17±5.53	49.7±2.45	64.16±15.42	69.56±17.72	65.75±4.39	63.42±3.29	61.35±9.57	80.09±26.47	52.3±1.27	74.86±10.10	86.33±50.40	69.98±7.2	55.70±0.31	66.86±16.6	108.60±21.16

1000192-63-3	530429	22.88±2.78	16.15±1.68	21.69±5.11	21.93±4.54	23.92±0	20.82±0.48	23.41±0	24.13±7.42	18.43±0.95	23.65±3.37	41.53±0	19.12±0		21.26±5.12	26.87±0	
629-62-9	12391	5.99±0.26	4.72±0.16	4.36±0.14	6.75±2.65	6.50±1.66	5.53±0.71	6.37±0	5.14±0.88	6.78±2.63	3.75±0.17	6.79±1.01	7.61±4.70	6.22±0.59	4.27±0	5.92±1.48	6.43±0.56
593-45-3	11635	3.90±0.54	6.41±0.17	2.95±0.57	4.63±0.64		5.16±0	4.11±0	4.54±0.90	5.60±1.61	4.96±0.83	5.94±1.31	5.95±3.40	4.42±0.38	3.63±0.17	3.82±0	7.92±0.86
544-76-3	11006	4.08±0															
629-99-2	12406	4.24±0		5.17±0.86	7.69±0		7.53±0.39		6.90±0.84			8.70±1.91	11.22±6.22	7.73±0	7.54±0.61	7.86±1.86	10.78±0
629-94-7	12403	6.06±0			6.12±0	8.64±1.96	4.75±0	5.79±0			10.53±0					6.63±0	
1000192-65-0	543423	4.15±0									3.15±0			1.89±0			
14905-56-7	85785	1.86±0			4.00±2.63	6.63±0		7.94±0	4.50±0	8.38±0							
55282-12-7	292285	0.84±0	1.83±0.11	1.01±0		0.99±0	0.78±0	0.71±0		0.89±0					1.11±0	1.69±0.55	
55000-52-7	41282		29.76±0.79			22.14±0		17.19±0				12.25±0	23.25±0	17.33±0		35.72±0	
629-	12401		8.74±0			9.37±0		6.35±0		8.18±2	7.18			8.34±0			17.84±

92-5			.59					.22		.23	±0						0
7390-81-0	23872		2.38±0				2.43±0			1.76±0	5.24±0	0.94±0				4.24±0	17.79±0
16489-32-0	565628	11.45±0		5.81±2.01	5.88±0		6.15±0	9.89±0		4.12±0.06	6.43±0.68	5.18±1.53	4.57±0	7.75±2.71		5.00±2.23	5.39±0
15780-34-4	162512			3.79±0						1.41±0	1.63±0					2.05±0	
55044-09-2	583842			3.99±0				4.51±0		2.71±0		2.13±0.61					
1000190-92-9	601922				9.39±0	5.67±0											
1000223-00-7	535067					10.48±0							16.99±0				
53818-14-7	143098						1.88±0							4.6±0	1.96±0		
294-62-2	9268						0.72±0					0.85±0					0.95±0
21328-57-4	524413						0.75±0										
16883-48-0	6429398						0.91±0										
54833-23-7	41208							5.33±0									

55668-09-2	544746							1.18±0									
112-95-8	8222								0.97±0				3.06±0				
1000156-14-5	572863												1.01±0	1.13±0	1.08±0		
1921-70-6	15979														18.05±0		
1000360-41-5	91693084														4.26±0		
57366-09-3	549006																3.36±0
2579-04-6	5364555	18.40±2.12	22.43±0.32	11.09±1.82	18.55±4.93	20.03±5.62	18.39±1.93	15.46±4.61	17.16±2.83	22.92±9.08	13.69±0.04	20.93±3.05	23.46±15.22	18.54±1.16	13.47±0.53	16.43±3.32	26.43±2.17
1000131-11-8	5364560	15.32±4.68	23.59±1.49	15.17±0			21.76±0	20.04±0	16.83±0				27.11±14.59	19.38±0	15.37±0		
18435-45-5	29075	3.00±0		13.76±0	20.55±3.72	24.81±5.02	24.82±0	22.43±0	25.54±0	27.74±6.95	19.91±1.33	27.55±5.64	7.96±0	23.85±0.25	20.88±0	15.08±12.48	36.83±9.94
33156-93-3	6440990	8.02±0	6.11±0.78	8.76±2.63	9.09±2.69	5.80±0	6.62±1.63	10.79±2.25	7.64±0.44	9.59±2.26	10.19±1.04	11.15±1.74	8.71±0	11.69±3.07	8.70±2.02	9.56±3.18	15.54±3.57

7683-64-9	638072	1.37±0						1.53±0		2.54±0							
22735-58-6	556470	2.92±0.8	2.31±0	1.13±0.25	2.65±0	2.10±0		2.35±0.55		2.55±0		2.62±0			2.48±0		2.51±0
89128-14-3	5372358	0.88±0.18		1.02±0				0.82±0			0.69±0			0.87±0.24			
16631-62-2	5367455	0.86±0				1.16±0	0.82±0.06					0.90±0			0.84±0		
1000131-11-6	5362676	19.64±0															
111-02-4	638072	2.08±0	1.68±0.12	1.25±0.03	1.61±0.17	2.00±0.46	1.77±0.07	1.55±0	1.64±0.17	1.34±0	2.06±0.56	1.92±0.4	2.49±1.52	1.42±0.05	1.38±0.08	1.29±0.27	2.50±0.04
2539-75-5	107833	1.70±0												1.29±0			
1000150-40-3	5367478	2.12±0		0.89±0.13	1.37±0.37	0.94±0	2.09±0.98	1.78±0	1.68±0	1.29±0	1.52±0.16	1.51±0.64		2.09±0.44	1.53±0	1.36±0	2.65±1.19
55449-71-3	41447		9.35±0														
10482-53-8	5352710		1.33±0						6.61±0								
54344-82-0	580068		1.15±0														
943-	6025413		8.27±0														

93-1																
1000194-37-0	605926	2.17±0														
26456-76-8	5365055		1.23±0		1.10±0		1.65±0.32	1.40±0	1.37±0.28	1.89±0	1.60±0.34	1.74±0	1.69±0	1.60±0	1.01±0	1.27±0
1000150-40-1	5367478			2.01±0				2.31±0								
56318-84-4	15607494				1.05±0						1.32±0					
56134-03-3	5352709					9.45±0									7.02±0	12.01±0
3983-06-0	138092					0.73±0									1.33±0	
100-42-5	7501						1.05±0				0.93±0		1.45±0	0.90±0	1.00±0	
74752-97-9	5368955							0.94±0.19								1.15±0
62185-56-2	545898									1.35±0						
54344-61-5	171338									5.94±0					7.30±0	
591-48-0	11573										1.07±0					
35387-	520731										1.39±0		2.02±0			

63-4																	
40467-04-7	545914												1.47±0	2.30±0			
6971-40-0	5365022													1.81±0	0.98±0		
5409-55-2	76420	1.73±0	2.90±0.21	2.11±0		2.69±0.95	2.57±0		1.95±0.65	1.44±0	2.02±0.10	2.31±0	3.25±1.48	2.91±0.04	2.47±0.12	2.41±0	4.99±1.82
98-86-2	7410	6.96±1.9	9.71±1.08	4.50±1.56	4.23±1.22	3.70±0.72	3.28±0.98	4.51±0.87	3.74±0.16	4.15±0.99	5.09±1.13	3.60±1.84	5.06±1.15	4.18±1.30	3.25±0.93	4.08±1.70	7.02±2.86
10396-80-2	146102	12.06±2.95	4.02±1.46	9.32±2.40	10.10±2.92	9.41±1.54	10.35±0.16	11.00±2.04	9.38±0.51	10.51±2.04	11.57±0.79	13.21±2.44	15.44±6.23	14.42±2.50	11.00±2.70	11.13±3.43	14.02±0.61
1000155-88-9	556457	1.97±0.61	1.55±0.52	1.04±0			1.46±0			1.03±0	1.37±0		1.28±0		1.66±0	1.38±0	2.51±1.01
2437-62-9	270491	1.22±0.23	1.67±0	1.26±0.34	1.28±0.67	1.43±0.56	1.50±0.04	2.03±0.05	0.78±0.01	1.92±0.77	1.69±0.14	1.98±0.26	1.91±0.50	1.54±0.29	1.37±0.24	1.64±0.63	2.97±0.84
52978-85-5	579763	1.72±0.59	1.97±0.60	1.33±0.4	4.89±1.80	2.02±0.23	2.32±0.33	2.25±0.10	2.91±0.54	3.35±1.47	1.70±0.03	2.24±0.11	3.29±1.91	4.01±0.52	3.11±0.59	2.34±0.08	5.42±0.76
765-69-5	13005	1.74±0	1.28±0.11				0.80±0										
10001	565306	7.20±0				7.27±0		5.17±0	4.41±0								

85-87-5																	
14353-72-1	586396	2.39±0				2.43±0	4.20±0.06		4.22±0.63				6.61±0		4.2±1.21		
1072-83-9	14079	1.38±0															
3796-70-1	1549778	1.02±0															
92-13-7	5910	11.25±0															
93-55-0	7148		2.09±0.39		1.46±0						1.85±0	1.27±0.22					3.31±0
38284-27-4	5352876		13.44±4.00			8.10±0.68	7.81±1.28	8.29±0	7.79±1.14	9.34±1.13	13.59±2.26	12.77±1.31	9.11±1.09	9.56±3.77	4.53±0	24.37±14.33	
83-33-0	6735		1.18±0														
80387-31-1	5368916		3.19±0							2.06±0	2.66±0	2.10±0					3.59±0
119-52-8	95415		1.89±0														
207669-70-3	543411			8.22±0					6.96±1.17	5.20±0		5.33±0			7.13±1.60	7.41±2.58	9.30±0
78210-68-1	529437				5.25±1.60									5.80±0		4.12±0	6.87±0

1000155-88-8	556457	1.49±0.39						1.34±0	1.57±0	
5870-63-3	138916	0.60±0		1.64±0						0.68±0
7370-92-5	110977		3.04±0							
1000304-66-3	1845939				4.50±0	4.12±0				
66884-74-0	75953512				3.71±0					2.29±0
26942-62-1	539006					0.81±0				
22118-01-0	572612						1.95±0			
4884-24-6	21003							0.78±0		
77573-35-4	101288159							0.76±0		
502-69-2	10408							0.94±0		
124899-17-8	573565								1.63±0	
58879-40-6	337818									1.82±0

22476-61-5	214839														3.92±0		
30316-33-7	594912																3.59±0
57-10-3	985	37.87±18.52	46.42±7.93	38.32±10.48	32.42±0.34	49.40±4.22	57.05±4.07	43.86±8.07	47.12±3.93	43.91±4.51	63.16±9.58	50.11±16.54	72.45±18.12	69.63±16.07	60±18.69	48.84±16.46	111.98±65.84
373-49-9	445638	12.19±6.06	12.22±2.00	14.42±4.14	14.38±1.02	22.93±1.59	25.37±1.65	16.05±0	22.07±2.06	20.77±2.63	29.73±5.48	21.90±7.38	24.50±0	32.54±6.90	39.13±0	22.52±6.51	76.49±0
64-19-7	176	23.79±12.89	6.29±0	20.69±0	5.35±0	13.18±0	34.90±0	40.73±3.79	24.92±12.56	12.59±4.33	10.08±0	32.46±15.95	23.65±0	41.26±0		18.68±0	
107-92-6	264	0.63±0	2.61±1.24	3.77±0	0.94±0	2.45±0.92	1.89±0	2.19±0.48	2.06±0.57				4.13±0	1.72±0.17	2.13±1.10	0.86±0.16	
544-63-8	11005	29.73±15.50	26.05±7.57	25.39±8.73	24.86±4.05	40.10±5.23	42.59±2.90	35.54±4.72	35.09±3.42	35.44±4.94	49.05±7.34	39.28±12.55	52.03±16.70	47.97±11.69	39.19±16.32	39.59±13.44	77.64±36.34
34450-18-5	1449	2.60±0												1.64±0			
1002-84-2	13849	3.70±1.63	3.33±0	3.38±1.14	3.03±0.13	5.45±0.68	4.39±0.18	3.60±0.35	3.86±0.51	3.75±0.76	6.59±1.43	4.85±1.99	5.56±1.99	5.68±1.26	3.39±0.35	3.91±1.45	8.31±3.62
463-40-1	5280934	5.53±3.15		1.41±0.24	3.34±0	2.04±0.85	1.87±0.43	3.03±0.65	1.70±0.19	2.75±0.64	3.13±0.26	3.97±0.74	2.45±0	4.57±0.4	3.20±0	1.47±0	3.32±0

112-80-1	445639	1.16±0.60	2.45±0.96	3.00±1.30	1.70±0	2.86±1.26	3.44±1.95	1.33±0	5.43±0		5.19±0.56	3.54±1.65	6.91±0	4.50±2.15	5.29±0	2.33±0.92	1.87±0
79-09-4	1032	2.35±0		1.15±0.30	1.74±0		1.55±0	0.77±0	1.74±0.65		3.47±0	1.04±0		2.34±0	2.57±0		
10417-94-4	446284	0.00±0.00	3.53±0	0.96±0	2.00±0.12	1.25±0		1.75±0		0.89±0	1.83±0	2.42±0	3.00±2.07	2.45±0	1.22±0	2.47±0	2.48±0.71
4536-23-6	20653	0.92±0						0.77±0				2.56±0				0.89±0	
6217-54-5	445580	4.79±0	1.00±0	5.39±1.50	4.37±3.07	5.41±3.88	7.04±0.46	9.41±1.56	4.89±3.16	7.64±2.87	7.90±1.45	6.03±4.15	9.80±5.22	5.25±1.05	7.21±1.42	5.90±4.12	16.39±6.13
143-07-7	3893	0.85±0	3.23±0	2.76±0		3.94±1.47	4.98±0			2.62±0	4.34±0.58	4.73±0	4.38±1.07	3.58±0.97		5.76±0	4.84±0.77
109-52-4	7991	43.92±0					1.70±0	3.15±0				0.85±0				2.02±0	
503-74-2	10430	1.61±0			1.20±0			0.77±0	1.44±0		1.24±0	1.14±0	1.72±0	3.78±0	1.26±0	0.89±0	
103-82-2	999			1.85±0	1.76±0	3.67±1.02	5.10±0.63	4.15±1.88	6.18±0				3.37±0	4.10±1.54	7.27±2.37	2.06±0	3.55±0
603-79-2	11782			0.76±0										1.07±0			
57-11-4	5281			0.73±0		0.73±0					0.95±0.0	1.31±0					

									2						
3420-36-8	99116658	0.94±0													
29743-97-3	5312435		1.15±0												
632-46-2	12439		0.83±0		0.97±0	0.76±0	0.97±0.03								
506-26-3	5280933			1.29±0	1.06±0			0.78±0				1.49±0	1.19±0		1.88±0
1000336-66-8	5282754				3.74±0		3.23±0								
638-53-9	12530				1.47±0										
2091-29-4	5282745					23.00±0					47.28±0		20.37±0		24.78±0
105-43-1	7755					3.81±0			0.76±0						
116-53-0	8314					0.98±0									1.03±0
1000316-61-9	6420405					1.96±0									
142-62-1	8892						0.80±0								
7561-	5312428												1.68	3.00±0	

64-0															±0		
16833-54-8	5312495														7.23±0		
56554-89-3	5367669				1.58±0						0.65±0						
100-52-7	240	49.51±10.75	61.44±2.56	33.89±7.22	39.62±7.17	32.99±3.70	41.19±4.59	37.33±5.93	34.00±2.45	29.63±8.31	29.52±3.93	27.00±5.68	42.90±13.48	35.94±5.23	23.98±1.94	29.65±8.54	59.95±23.45
122-78-1	998	4.75±0.76	6.4±0.4	4.18±1.01	6.20±1.64	4.73±0.91	5.97±0.58		2.90±0.89	3.54±1.39	6.28±0	3.85±1.28	6.65±0	6.66±1.33	7.87±0	4.32±1.19	6.66±0.79
1000130-97-9	5363097	4.49±0	7.35±0.81	3.31±0	7.13±0												
57295-30-4	585182	5.45±0															
56829-23-3	5365665	16.10±4.45		11.57±3.29	10.82±2.18	8.91±1.22	9.68±0	11.71±3.33	10.64±1.17	7.33±0	12.06±2.66	10.88±1.42	13.55±3.17	15.32±3.00	15.32±0	12.22±0	
4411-89-6	6429333	1.50±0.38	1.29±0	1.16±0.34	1.59±0.39	1.03±0.02	1.52±0.28	1.91±0.27	1.89±0.34	0.70±0	0.91±0.01	0.84±0.11	0.80±0		2.31±0	0.98±0.24	1.74±0.31
53447-47-5	71587170	8.32±2.13		4.85±0	8.04±2.10	7.12±1.65	6.33±1.54	7.37±0		8.55±0		8.15±0	12.59±0				
629-80-1	984	3.59±0	8.67±2.1	3.01±0	8.78±0.57	6.56±0.33	6.28±2.70	4.47±0.29	5.71±0.92	5.69±0.31	9.66±2.38	5.98±3.24	5.65±2.02	4.50±0.07	2.27±0.37	8.19±4.23	18.07±9.17

124-19-6	31289			1.34±0.39	1.31±0									3.55±0			
111-71-7	8130	1.24±0							0.72±0					1.89±0			
5910-87-2	5283339	1.54±0					1.19±0							1.25±0	0.85±0		
4313-03-5	5283321		2.70±0.56		4.29±0.02					4.50±0	3.17±0.70	2.98±0.46				3.06±0.07	3.69±0.84
931-96-4	565407		5.97±0		4.68±0	5.50±0	6.10±0			5.12±0		2.10±1.48	11.60±0	13.22±0	10.04±0	5.54±1.61	7.97±0
23942-00-9	585875		6.48±0														
4748-78-1	20861		3.46±0	2.08±0	3.11±0.47	2.63±0.23	2.66±0.19	2.35±0	2.57±0.45		2.57±0.51		2.65±0.47	2.43±0	1.74±0.48	1.04±0	3.96±0.98
41438-18-0	458185			4.24±0	2.35±0.99		1.82±0			3.56±0	2.98±1.19	4.28±0				4.26±0	2.96±0
85896-31-7	522841				2.16±0		2.47±0			2.14±0						2.13±0	
30361-28-5	5283329				1.24±0.38	1.39±0		1.28±0		1.22±0				1.03±0			2.10±0
557-48-2	643731				1.86±0							1.65±0					
2423-	5364492				1.15±0						3.18						

10-1											±0						
20235-19-2	854						1.23±0										
21834-92-4	5370602							2.19±0			2.46±0						
1000461-11-8											1.52±0			1.95±0			3.12±0
56554-87-1	5367668											1.77±0				1.21±0	
1196-67-4	5372857													1.25±0			
63521-76-6	544162														2.82±0		
500-66-3	10377	4.68±1.78	6.78±0.12	5.10±0.52	8.11±2.08	5.26±1.18	7.76±0.54	6.20±0.24	8.37±0.40	9.60±3.38	6.06±0.30	8.09±0.66	7.71±3.65	10.52±0.56	7.86±1.23	9.08±1.88	13.65±1.34
3391-86-4	18827	1.20±0		1.18±0	0.60±0						0.82±0						
5353-25-3	5364713	2.17±0.92	2.99±1.60	3.74±0	4.38±0.36	1.94±0.05	1.92±0.68	1.75±0	1.52±0.53	1.55±0.37	4.78±2.03	1.21±0	2.01±0	2.13±0		1.28±0	5.83±0
1855-09-0	15825	2.83±0	3.78±0							1.33±0	1.71±0						
18240-10-3	86682	10.71±0		5.68±0			5.17±0	5.68±0					7.35±0	7.29±0			

22117-09-5	5367371	2.14±0			3.39±0			2.82±0		3.18±0	3.65±0	3.08±0		3.65±0		3.99±0	5.25±0
30385-19-4	545738	3.58±0.34		2.56±0	1.09±0				0.88±0								
1454-84-8	80281	4.22±0	4.21±0.22	2.23±0			3.02±0	3.25±0	2.92±0		2.58±0.01		1.26±0		2.53±0		5.02±0
24347-58-8	225936	3.73±0				2.57±0	2.42±0	0.93±0					1.39±0		4.95±0	0.88±0	
766-00-7	69833	3.84±0		2.56±0													
468-68-8	3080551	2.17±0	1.06±0														
34492-42-7	5368564	2.01±0		1.66±0							1.75±0						
6728-31-0	5362814	1.34±0															
1075-04-3	15825		2.50±0		2.29±0	1.19±0						2.17±0					
1464-44-4	65080		1.89±0.49														
1000193-38-7	565579		3.90±1.42				4.67±0						9.09±0		2.63±0		8.45±0
17289-61-1	11579182			1.08±0													

96-07-1	95331	0.78±0							1.43 ±0					
698-87-3	94185	0.76±0												
1000196-32-3	565522		7.77±0	6.52±0			5.39±0.45	8.99±0	7.52 ±1.60	8.99±0		4.28 ±0		7.44±0
90970-69-7	114544		1.20±0							1±0				
35727-45-8	101316861		0.91±0											
5181-75-9	291783		0.93±0	1.82±0.25			0.98±0	1.81±0			3.44±0		0.68±0	1.60±0
56045-33-1	556415		1.37±0											
117221-78-0	556791		1.77±0											
1000187-22-4	580092			0.80±0		1.41±0								
1000184-98-8	561943			2.54±0	3.21±0			4.70±0		2.41±0			3.33±0	5.87±0
119660-66-1	608998				1.98±0					1.71±0				
655-	95447				1.35±								1.20±0	1.16±0

48-1		0						
53404-49-2	62044	1.22±0						
1454-85-9	15076		4.99±0					
1000129-79-9	560124		0.74±0					
51937-00-9	6436736			7.20±0				
23261-20-3	15942827			1.31±0.29			0.98±0	
7212-44-4	5284507			1.22±0				
1000131-11-4	5364768				1.09±0		1.42±0	1.17±0
105794-58-9	537071				3.89±0		2.60±0	
17367-07-6	5367702					2.33±0		3.77±0
90676-25-8	138614					3.07±0		
7735-42-4	82184					3.19±0		
4621-	20739					1.31±0		

04-9																	
1000130-07-6	345716											1.26±0					
1000128-40-9	242517													1.73±0			
2490-48-4	17218													4.18±0			5.52±0
77611-58-6	560936															2.45±0	
1000131-10-9	5283296																5.91±0
33425-30-8	87985					16.11±0				29.16±0							
1000222-86-6	9602988	5.19±1.02	19.49±4.99	5.13±2.68	12.21±8.06	8.64±4.93	5.34±1.52	2.09±0.52	6.55±3.75	9.90±1.67	10.89±6.80	12.24±3.55	11.27±4.04	8.17±1.32	1.27±0.16	7.79±4.55	53.68±37.18
131-11-3	8554	1.55±0.48	1.42±0.27			0.85±0	1.55±0	2.12±0.41	0.72±0	0.80±0	1.08±0	1.10±0	2.25±0.54	2.29±0.41	2.21±0.49	3.52±2.00	7.30±2.82
24691-16-5	229657	1.11±0			1.14±0	1.05±0	0.97±0	0.72±0	0.75±0						0.80±0		
2566-90-7	6421262	0.68±0									0.93±0						

112-23-2	8169	1.80±0.33		1.23±0	0.63±0			0.74±0			0.70±0.08	2.73±0		0.90±0	0.97±0		
2716-53-2	5364833	0.70±0			1.82±0	3.15±0		3.13±0.57		3.21±0.04			3.29±0		2.11±0		9.49±0
76337-16-1	637195	1.65±0							2.33±0								
1499-53-2	226558	1.57±0									1.45±0						1.93±0
80-27-3	62328	5.22±1.48															
10236-16-5	637195	2.18±0	4.17±0.71	2.38±0.91	3.28±0.1		3.14±0.15	2.72±0.19	2.60±0	3.06±0.74	4.43±1.72	3.22±0.85	4.28±1.63	3.59±0.88	3.82±1.57	3.45±1.67	7.28±3.47
628-97-7	12366	1.63±0	1.15±0	1.33±0	1.13±0.05	1.72±0	1.63±0.27	1.24±0	1.53±0.08	1.24±0	1.97±0.60	1.32±0.51	2.25±0.85	1.80±0.19	1.75±0.63	1.15±0.16	2.60±0.94
112-39-0	8181	0.78±0					0.78±0					0.71±0		0.72±0	0.74±0		
55724-48-6	101287780	1.09±0													1.81±0		
17092-92-1	6432173	0.92±0															
89-48-5	27867	4.42±0														2.58±0	
28474-	168814	0.84±0				1.23±0					1.32				0.85		

90-0											±0.36				±0		
77508-64-6	101280823	1.19±0				1.20±0			1.00±0						1.30±0		
1000336-36-7	14122946		1.40±0														
1000131-33-2	5363633		2.15±0									1.11±0					3.52±0
10605-09-1	54725318		1.04±0						0.75±0								
1000305-65-2	6422010			2.67±0		2.85±0		4.38±0					10.83±0				
1000330-54-1	5461492			2.25±0	2.99±1.2			3.61±0	3.61±0.48	4.94±0				3.87±0			
1000466-87-1	165361954			0.75±0													
1000130-99-3	5363221			0.82±0										1.12±0			
1000130-97-	5365029				1.33±0												

4														
1000130-99-6	5363222	1.57±0		1.79±0										
5487-50-3	554151	0.83±0						1.13±0.55	1.01±0				1.03±0	1.34±0
3766-81-2	19588	0.93±0	1.16±0			1.53±0								
16409-45-3	27867	2.37±0												
32025-37-9	595877		1.63±0											
1000406-16-0	91693199		1.48±0											2.68±0
504-40-5	101269			0.76±0										
1000129-71-8	570737			7.92±0.52										
1000132-45-6	5365020			4.63±0										
5680-83-1	72771				5.41±0					3.73±0				

1000336-80-1	91697555	1.27±0									
713-95-1	12844	18.42±0									
761-35-3	99931		0.73±0					1.16±0			
710-04-3	61204		8.54±0						7.51±0		
1000253-26-4	569848			4.13±0							4.48±0
1000131-31-1	539322				0.79±0						
22393-88-0	6436542				0.67±0						
56309-62-7	4178425				1.31±0						
1000210-45-4	556352					1.43±0					
75405-43-5	13816515					4.01±0	3.61±0				
5431-33-4	5354568					0.90±0					

1000131-36-3	5367730											1.96±0					
56687-67-3	534623													1.80±0.82			
84494-70-2	5353613													1.08±0			
1000391-10-5	91695146													1.00±0			
1000128-67-1	536834													7.84±0			
22522-34-5	5365040														0.82±0		
107-43-7	249															1.20±0	
62555-02-6	71385260		2.30±0														2.59±0
5637-97-8	3639916								1.80±0		1.37±0						
1000130-04-4	541568				1.06±0								1.53±0				
128-37-0	31404	49.31±6.49	28.00±6.35	38.91±5.17	46.75±13.77	48.04±9.65	52.75±1.91	48.91±3.71	45.68±2.31	56.82±18.90	42.30±1.	54.45±4.59	73.50±36.7	62.68±9.13	51.99±8.	47.37±10.43	66.41±1.24

										97		0	78	
2896-60-8	17927	1.24±0.47		0.96±0						1.09±0	0.93±0			
1126-61-0	70775		1.16±0		1.20±0	0.93±0.18	1.19±0		1.22±0	1.14±0		2.06±0		1.25±0
2416-94-6	17016		1.15±0											
3855-26-3	77462					4.02±0								1.65±0
3180-09-4	18515									0.74±0.09				
4025-37-0	97697									0.83±0				
1000152-81-2	6432249									2.63±0				
1000337-61-7	5306963									1.56±0				
104-63-2	4348									4.63±0				
108-45-2	7935								1.98±0					
134001-62-0	547067							0.97±0						

1000338-20-2	11298049					4.77±0							8.86±0				
124045-56-3	578315				5.97±0												
1000111-25-6	585061			3.47±0													
30950-27-7	5365782		1.72±0														
1000296-66-8	578435		2.88±0														5.19±0
631-61-8	517165		1.67±0	21.19±0	16.94±0									50.29±0	41.33±0	57.02±0	
75-50-3	1146	1.66±1.02		2.10±0			1.09±0	0.82±0	0.73±0	1.23±0							
3102-70-3	76547	1.32±0.32	0.97±0	1.12±0				1.07±0			0.7±0	0.95±0.16	0.67±0	1.32±0			
296245-21-1	581431	1.33±0.02	2.72±0	2.93±0		2.60±0.71	3.12±0.12	3.20±0		1.83±0	2.44±0.29	2.39±0.59	3.39±1.42	5.34±0	3.11±0.69	3.64±0.97	4.34±0.13
529-19-1	10721	0.83±0															
1000185-13-	567280			1.61±0	3.46±0	1.85±0.22				2.34±0			2.71±0.72	2.27±0			1.94±0

3																	
1000303-08-5	6422106							1.86±0	1.84±0					1.03±0			
140-67-0	8815	1.09±0	1.08±0	0.73±0	1.60±0			1.42±0.21	1.24±0	0.82±0	0.72±0		1.37±0.4	1.89±0.23	1.01±0.09	1.43±0.47	1.76±0
538-86-3	10869							3.33±0							3.04±0		
104-46-1	637563																3.34±0
622-60-6	73955																2.45±0
54060-30-9	104682		6.18±1.32							4.67±0	7.29±1.01					8.50±0	16.65±0
1000150-17-9	546887									1.29±0							
74744-55-1	5367448											2.54±0		2.40±0			
16695-32-2	86051		3.67±2.56												3.56±0		

Imported fishmeal

CAS	Compound	Relative content (μg/kg)															
		0d	2d	4d	6d	8d	10d	12d	14d	16d	18d	20d	22d	24d	26d	28d	30d

	CID																
91-20-3	931	38.21±15.24	34.87±13.32	32.28±4.81	69.76±30.92	40.22±7.97	30.49±1.00	27.54±1.22	29.48±4.68	61.91±12.21	33.51±6.95	24.43±4.51	46.40±9.01	41.00±1.97	54.85±19.18	60.08±15.92	67.25±13.67
91-53-2	3293	419.28±141.14	371.40±166.29	252.53±25.70	496.32±125.00	450.30±121.49	308.02±11.87	268.49±0.82	269.23±21.23	400.79±44.81	322.04±120.47	146.32±2.87	274.95±81.78	187.25±8.83	334.30±115.30	275.02±58.02	353.73±16.17
1008-88-4	13886											3.10±0	5.11±2.13	4.82±0	9.99±4.06	11.32±0	9.67±4.36
91-57-6	7055	8.55±3.29		8.68±0		11.76±2.65	9.49±0.72	8.46±0.37	7.64±1.30	26.58±2.55	10.57±2.84	6.49±0.23	14.89±1.74	13.88±1.19	22.90±7.96	15.88±4.05	29.70±5.95
3208-16-0	18554		4.92±0	4.42±0	9.43±0		4.50±0	2.37±0		3.56±0	2.14±0		3.29±0		3.55±0		
59958-20-2	181250	4.99±0		5.27±0													
16489-90-0	86013	46.13±18.56	46.55±16.36	29.36±7.55	55.69±15.29	37.76±10.8	29.79±1.94	28.81±2.21	35.44±7.42	40.51±6.46	31.38±10.32	15.64±2.32	32.13±6.42	20.46±0.57	36.35±12.83	29.14±0.99	43.69±2.27
18108-56-0	614744	15.08±3.61	4.58±0	8.68±0		27.28±8.01	9.55±4.84	7.89±4.38	3.82±0	13.94±5.57	16.56±4.96	18.65±0					
93008-87-8	617681	16.92±6.60	7.40±0.83	6.82±3.20	20.52±8.16	18.10±0	8.39±0	8.36±2.94	8.55±3.25	24.23±17.51	9.57±1.07	29.37±2.49	30.83±6.97	44.73±4.32	48.21±11.69		39.45±15.36

147-47-7	8981	3.10±0	4.54±1.48	2.62±0	5.13±0		3.62±0										
65539-79-9	5374980	2.71±0	4.30±0.8	3.84±0	3.94±0	6.19±2.50	5.36±0.18	4.82±1.13		4.18±0							4.35±0
70424-13-4	5370006		8.57±0	8.40±0.01	7.59±0	3.41±0	13.00±0	3.79±0		6.67±0	3.89±0		2.68±0	4.27±0	8.95±1.26		6.85±0
23095-44-5	96943					20.70±0	4.30±0					4.71±0			27.39±0		27.98±0
84679-58-3	614550					13.76±0				4.02±0			3.16±0	4.41±0	6.79±2.71		5.91±0
54964-85-1	281839					11.07±0											
46873-19-2	618028								11.85±0								
3606-26-6	615798	5.62±0							8.37±0								
132-64-9	568									6.29±0						10.64±0	
582-16-1	11396									6.21±0							

827-54-3	13230									4.94±0							
1000337-86-4	16761950	9.85±0							3.22±0	3.56±0	14.42±0		11.45±0				
151-10-0	9025											3.23±0	7.32±1.56	4.70±0	9.75±3.39	6.11±0	13.70±3.11
3905-64-4	77509																14.29±0
92-52-4	7095									4.19±0							5.52±0.91
571-58-4	11304																3.80±0
52823-95-7	301207		23.32±0					27.36±0	31.21±0								
1000156-41-6	614939				24.93±0		7.73±0										
1000129-30-2	617842			7.92±0													
120-72-9	798															11.19±0	

629-78-7	12398	5.26±0.35	6.34±0.20	6.19±1.41	9.70±0.28	22.67±5.01	15.56±2.30	15.27±0.63	8.33±2.13	13.97±1.07	15.17±4.83	8.15±0.84	16.72±4.99	13.82±1.81	25.27±7.38	10.92±0.28	23.10±0
1000192-63-3	530429	22.79±0.39	19.68±0	42.59±0		34.70±0		20.41±4.28		1.78±0	25.13±0						
629-62-9	12391	5.15±0	10.02±2.78	11.90±1.53	11.07±2.35	19.86±4.02	13.29±6.63	14.73±0.08	5.77±0.02	11.61±5.21	15.16±2.66	6.96±0	12.54±4.03	9.39±1.41	13.77±4.93	9.11±4.07	
544-76-3	11006					14.49±3.84	13.46±0	10.37±0.20	6.54±1.34	13.86±5.29	13.80±3.37	7.46±0	15.75±4.92	15.14±3.21	20.53±7.02	7.68±0	19.19±0
14905-56-7	85785			2.56±0												8.12±0	
55000-52-7	41282		30.16±6.71	31.82±0	37.21±0		49.33±0	42.82±0.96							23.27±0		
7390-81-0	23872	11.64±0		5.25±0		26.87±10.03	11.43±8.14	8.97±4.66	3.12±0	10.87±4.61	25.78±0		3.65±0	34.06±0	25.20±16.28	78.98±0	
1000190-92-9	601922	2.95±0	7.32±3.49	8.36±1.17	8.43±0	6.45±0	10.42±0	6.17±0									
54833-23-7	41208			4.77±0			7.08±0										

1921-70-6	15979					58.94±11.81			40.61±5.93	38.27±11.04	16.00±0	48.22±0	35.93±6.08	56.84±19.00	23.82±0	56.83±0	
1000150-98-9	617625	3.54±0	4.30±0	2.35±0	4.96±0	7.30±0	6.75±0.56	6.51±0.83	3.71±0		3.95±1.39	1.80±0					
24251-86-3	520105				4.37±0												
1560-96-9	15269						2.83±0										
1000192-21-5	5368226																15.25±0
638-36-8	12523																14.31±0
2579-04-6	5364555	5.08±0	6.89±0.86	4.95±0.50	8.17±1.32	14.56±3.95	10.00±1.72	10.96±0.28	5.42±0	4.97±0	3.20±0	5.73±1.35	4.78±0				
18435-45-5	29075			2.57±0			5.28±0	4.18±0									
33156-93-3	6440990	3.88±0	7.88±2.77	4.76±0.48	7.16±0	4.63±1.21	4.79±0	3.88±0.21	2.47±0								

16631-62-2	5367455			2.43±0				2.78±0									
10482-53-8	5352710													4.12±0	9.69±2.14		3.88±0
943-93-1	6025413			7.46±0						3.87±0		1.75±0					
1000150-40-1	5367478										5.74±0						
75581-03-2	5366013	7.55±2.35		5.29±0	7.88±0	19.55±7.15	6.33±3.03	4.99±0.05	6.45±0	28.20±0	8.65±0	3.65±0	10.80±0	23.29±0			
137235-42-8	595401		11.07±3.34	7.27±0	9.72±0												
264-09-5	637504		5.97±2.40	7.22±0	13.9±5.74												
106-87-6	7833			2.30±0	5.34±0												
16631-66-6	5367476						4.31±0										

61142-06-1	565944								6.12±0		14.02±0			8.52±0			
1139-30-6	1742210												3.63±0				
26266-08-0	5366021		2.61±0														
591-49-1	11574													4.02±0			
5409-55-2	76420	13.37±3.27	17.38±5.57	10.93±0.72	20.58±6.25	17.71±3.82	13.65±1.51	14.46±0.38	10.72±2.19	14.85±1.01	9.91±3.10	5.14±0	10.67±1.96	6.70±0.42	12.29±4.20	9.35±1.05	18.77±0.77
38284-27-4	5352876	5.74±1.04	9.05±4.10														
207669-70-3	543411																3.77±0
77573-35-4	101288159					5.03±0						9.56±0		29.91±0			
1000349-85-9	3065113	276.73±152.22	197.24±75.85	154.33±34.09	380.61±190.41	201.72±25.25	157.22±12.70	142.12±8.21	138.74±15.26	282.27±121.41	164.17±42.18	55.96±14.98	155.78±30.45	126.23±5.01	213.20±78.31	344.70±196.38	232.14±33.82

1000159-95-5	618020	32.15±5.39	28.43±12.96	18.61±3.25	44.81±15.97	62.26±14.02	32.41±9.80	27.64±5.19	24.12±1.20	37.76±5.98	40.34±17.57	10.43±3.08	24.75±0.08	21.10±6.26	62.31±16.09	29.30±6.70	49.40±7.57
82304-66-3	545303	6.91±0				10.96±0		6.83±0	8.73±0		6.95±0		13.16±0		41.24±15.91	24.20±0	28.69±0
5452-39-1	227363	5.86±0			7.34±0												
1000164-02-4	5369921	4.87±0	9.51±0	3.90±0	6.43±0.18		4.82±0										6.68±0.43
730-46-1	97747	10.34±5.54	2.38±0	4.30±0	6.39±0	15.34±5.98	4.67±2.03	3.52±0.57	5.18±1.05		25.98±0	6.97±0					
133727-45-4	620875	4.16±0				8.31±0											
1000217-12-6	592620	3.03±0	3.48±0														
112-12-9	8163			5.31±0									16.71±0	4.68±0	12.73±3.56	12.21±1.74	
6831-17-0	165536	5.77±0		6.15±0			5.43±0			4.55±0							
4727-18-8	543400					38.15±0											

1000132-12-7	5376129	24.63±0											
23747-14-0	90971	12.28±0		8.69±0			8.09±0						
30666-87-6	10921984	9.22±2.30	9.09±0	8.95±0.11	4.51±0.38	7.66±0	5.00±0	3.19±0	4.83±1.98		5.51±2.09		8.17±0
19598-45-9	177072	6.14±0	6.77±0										
29204-24-8	76099200				5.28±0				8.76±0	11.58±0	30.02±8.93	13.14±0	23.25±0
2345-28-0	61303												32.52±0
77646-23-2											4.59±0		7.37±0
26078-25-1	96929							1.53±0					
2922-51-2	18027										27.51±0		

57-10-3	985	242.44±111.45	169.96±74.20	118.87±9.50	326.37±99.43	207.77±0	133.36±19.11	139.56±0	87.66±25.69	241.08±167.50	110.55±98.99	81.06±2.30	381.66±213.60	389.93±0	376.30±126.84	688.25±122.94	468.50±117.40
373-49-9	445638				63.63±0	70.44±0		41.25±0			104.21±0	40.95±0	95.27±1.26	261.00±92.22	148.25±0		
64-19-7	176	19.56±0	46.28±0	14.73±0.45	26.54±0	13.03±0.53	16.24±0	9.20±6.41				1.73±0			9.03±0		
544-63-8	11005	40.24±2.63	46.23±28.81	28.35±11.59	80.47±24.44	155.05±46.67	21.20±10.33	45.06±16.34	44.37±23.67	40.35±22.80	62.26±3.40	38.56±3.12	101.60±38.39	95.17±29.11	404.83±133.38	152.73±107.12	285.72±39.29
1002-84-2	13849		7.08±4.19	2.75±0	14.47±0	31.70±23.28	27.92±0		6.45±0	4.97±0	29.11±9.39	4.11±0	18.45±12.29	9.26±0	82.31±49.95	18.85±0	21.04±0
112-80-1	445639	11.49±1.46		51.42±0	16.54±7.94	3.90±0	4.75±0.83	8.29±0	28.30±0	8.09±6.02	25.91±4.09	34.23±5.05	42.42±0	39.04±0	36.07±0		
10417-94-4	446284					11.13±0											
6217-54-5	445580	4.08±0	8.19±2.52	4.10±0.60	8.39±0	12.92±5.01	11.33±4.19	7.78±4.65	3.70±0.02	4.97±0		1.74±0					
143-07-7	3893	3.54±0	6.47±1.21		4.96±0	7.76±0		3.15±0.76									
57-11-4	5281				7.76±0												

2091-29-4	5282745	118.28±0	52.92±19.33	55.10±1.68	112.73±0		37.21±0		45.34±12.84	70.43±0	7.86±0	37.50±0	87.91±16.67	35.63±0	153.90±0		
20257-75-4	11162521	8.12±0	4.33±0			19.65±2.89	7.59±0	4.78±0	36.25±0	14.31±0	5.28±0	18.61±2.53	30.44±6.28	29.25±3.44			
1000192-71-8	495454	5.62±0	5.17±0	11.36±1.86	52.03±0		21.74±0	6.68±0		53.47±0							
1000195-87-5	5365892			8.08±0													
506-12-7	10465				4.19±0												
506-32-1	444899					23.42±0											
17735-94-3	5312518									44.90±0							
100-52-7	240	32.60±8.93	39.43±13.75	28.41±6.10	55.00±27.86	22.44±4.71	19.11±0.80	16.55±1.07	11.74±1.42	24.19±7.48	12.96±2.17	6.73±0.35	15.65±2.93	18.36±3.28	10.92±0	14.83±0.16	16.25±2.45
122-78-1	998				4.89±1.40										5.31±1.98		3.98±0
56829-23-3	5365665	4.77±0	11.60±4.67	10.07±0	12.35±0	7.52±1.30	8.63±0	5.98±0.62	3.46±0		4.14±0.38						

53447-47-5	71587170		6.60±0	3.42±0.45	4.26±0		3.44±0	2.86±0.05		3.44±0	2.20±0						
629-80-1	984	35.85±14.78	27.07±13.63	30.07±0	44.46±20.62	81.23±26.76	48.29±12.63	42.25±1.36	24.96±3.98	55.55±18.52	54.72±28.71	6.33±2.69	31.06±4.78	30.25±7.02	58.23±14.13	31.49±2.67	49.93±8.10
41438-18-0	458185		4.69±1.74	2.59±0													
85896-31-7	522841														57.53±0		
2423-10-1	5364492		4.98±0														
1196-67-4	5372857				3.48±0				3.48±0								
58594-45-9	5364497	37.56±24.20	14.54±10.04	16.16±0	20.14±0	37.54±0.14	32.32±7.60	22.72±0.28	13.66±0	87.66±0		9.11±0				81.68±0	
638-66-4	12533	71.98±0	12.27±3.91		58.24±20.93	32.66±5.20	20.41±0.32	24.40±2.81	59.17±46.58	56.63±25.70	20.26±4.57	61.99±8.83	83.30±23.87	169.92±0	137.59±15.69		
4997-36-8	617826		11.39±0		13.81±3.76												
101-39-3	5372813		4.07±1.64	2.76±0.27													

124-25-4	31291			2.18±0		6.24±0		3.30±0.1		3.22±0	5.42±0			4.08±0.01	5.76±0		
330207-53-9	5363106	5.95±0							6.24±0	4.44±0				3.74±0			
62238-34-0	71590			7.78±0											21.88±0		14.47±0
2765-11-9	17697																22.37±0
56219-04-6	5364643																9.69±3.74
500-66-3	10377		4.69±1.47	2.83±0.46	4.15±0		3.62±0	3.53±0.34	2.85±0.4	3.23±0	3.38±0.87	1.89±0	3.60±0		5.88±0		7.50±0
5353-25-3	5364713							11.78±0			16.66±0	1.47±0	23.17±0	6.53±0	103.60±0	21.92±0	
18240-10-3	86682		5.74±2.28	3.93±0													
1454-84-8	80281					10.74±0					6.31±4.13				7.73±2.31		
24347-58-8	225936			2.99±0	3.52±0												

766-00-7	69833													13.40±0	32.18±0		
17289-61-1	11579182				10.53±0												
1000184-98-8	561943					11.80±0	2.84±0	8.18±0		7.21±0	7.84±0						
119660-66-1	608998	18.13±0.77	34.77±11.15	23.43±0.29	28.90±3.45	33.21±6.54	23.14±6.09	29.25±0.01	16.16±2.30	21.58±6.10	22.06±4.88	10.97±0	20.40±5.85	13.43±0.56	24.55±8.73	12.96±4.50	28.47±2.22
1000131-11-4	5364768	18.63±0			13.38±3.91												
105794-58-9	537071					19.52±0			15.80±0								
17367-07-6	5367702	11.68±0		13.53±0	32.33±20.19												
2490-48-4	17218		2.96±0		4.16±0		5.67±0										
5240-72-2	21308	2.99±0															
506-43-4	5365682					78.25±0											4.87±0

68931-30-6	92862					29.32±0					33.16±0						
56554-77-9	557439	5.74±0	2.81±0			8.20±3.28	5.44±0.46	4.46±0.05	4.42±0	4.61±0	6.66±0	3.00±0				19.38±0	
112248-30-3	5364523									7.22±0			7.06±0		12.48±0		
1000404-81-9	250092											1.94±0					
6617-35-2	520984										4.18±0						
19894-97-4	88301												6.92±0				6.86±0
33425-30-8	87985								6.58±0			1.22±0					9.99±0
1000222-86-6	9602988	13.65±0		3.66±0	12.40±0	6.97±0	19.58±12.69	8.29±5.07	137.06±105.96	24.13±9.40	24.50±11.70	39.86±18.24	41.57±10.17	57.11±20.99	218.50±161.44	88.07±26.69	
2566-90-7	6421262	17.98±8.3	6.73±0.47	12.93±0	11.76±0	34.36±12.95	11.17±3.88	7.65±2.52	12.48±0	4.96±0	27.13±7.68	14.31±0	50.95±1.35	53.90±9.39	76.89±7.31		

2716-53-2	5364833							15.86±0			13.51±0	6.40±0			56.51±0		
76337-16-1	637195	20.16±0				58.13±46.60	17.99±0				4.45±0	17.71±0	18.17±0	72.23±24.18	34.85±0	37.74±0	
10236-16-5	637195	24.66±0	19.91±11.33	12.59±7.74	30.31±8.79	44.00±0	13.78±3.55	37.53±0	19.57±5.19	37.48±12.21	49.10±22.47	31.01±0	38.94±0				
628-97-7	12366	22.34±9.61	13.77±5.21	9.23±4.13	24.88±5.40		15.17±3.50	29.26±13.52	41.99±0								
55724-48-6	101287780										42.93±0		8.56±0				
28474-90-0	168814											7.93±0			19.38±0		
77508-64-6	101280823			3.03±0													
1000131-33-2	5363633				10.04±0		19.04±0			8.49±0		7.83±0					111.74±0
1000330-54-1	5461492											3.52±0					

1000130-99-6	5363222														5.91±0		7.23±0
56309-62-7	4178425							3.64±0									
84494-70-2	5353613				25.84±0					40.36±0		10.45±0				62.29±4.81	77.28±33.00
1000128-67-1	536834										2.61±0						12.43±0
22522-34-5	5365040				12.05±0												
124-06-1	31283	8.45±0	9.26±0	7.72±0	7.84±0	12.69±0	12.42±0	12.28±0		3.51±0	7.28±0						
22597-23-5	83352	3.09±0															
111-62-6	5363269			2.19±0		12.25±3.97	3.77±0		3.42±0	7.97±0	4.86±1.99						
1000368-24-7	346279			3.15±0													

54546-22-4	5364759	50.79±0	30.22±0						
693-80-1	5363289	19.74±4.39				115.82±0.46	108.38±0	13.93±0	
1000333-54-8	12823768	23.82±0		18.01±0					
1000405-21-1	87382954	165.27±0	62.06±0						137.95±0
61691-98-3	470667513		9.17±0						
1000211-50-5	615498			36.84±0					
6963-44-6	81414					6.35±0			
1000043-05-3	6452096						63.45±0		
28474-90-0	168814				7.93±0		19.38±0		

1191-16-8	14489									10.05±0		
118-60-5	8364			8.34±0							12.68±0	
83834-59-7	5355130			18.44±0								
118-56-9	8362			7.66±0								
51906-13-9	101643328				5.02±0							
1000130-81-2	5363512					3.88±0						
623-65-4	69339						112.47±0	42.83±0	178.84±0			
1000130-81-0	549821							13.04±0				
22393-98-2	19010216											73.26±0
134001-62-0	547067	5.49±0	2.10±0									

631-61-8	517165	4.97±0								14.13±0							
75-50-3	1146		37.77±0	10.24±0	5.53±1.26		2.86±0	3.42±0									
1988-88-1	137274	7.27±2.03	6.78±4.43	2.59±0	6.43±0												
1000404-82-1	66697	3.07±0	6.21±2.06	5.13±0				3.81±0	2.88±0.34		2.57±0						
101-40-6	7558				5.40±0												
1000192-22-5	5376991					39.86±0	13.42±0				24.78±5.22						
732-27-4	135476867								7.32±0		6.65±0	3.36±0			11.62±0		
298213-31-7	612020										15.58±0						
13022-04-3	618053													18.88±0	58.53±0		
1000191-54-4	597835													5.01±0			14.28±4.17

1000337-58-3	5306773			4.18±0													
140-29-4	8794																4.09±0
54060-30-9	104682	7.78±2.96	10.32±4.40	8.29±0		5.07±0	4.09±0	6.17±0	4.20±1.03	6.84±0	9.55±0	1.37±0					

Table S2 The results of the significant correlation coefficient between freshness indexes and VOCs

(Domestic fishmeal)

Domestic fishmeal					
CAS	Compounds CID	Correlation coefficient and significance			
		AV	pH value	VBN	Mold counts
128-37-0	31404	0.660**	0.477	0.392	0.427
91-53-2	3293	0.292	0.524*	-0.139	-0.165
500-66-3	10377	0.552*	0.342	0.594*	0.714**
1008-88-4	13886	0.651**	0.417	0.624**	0.653**
90-12-0	7002	0.401	0.529*	0.477	0.542*
95-20-5	7224	0.559	0.804	0.691	0.941*
13067-27-1	83101	0.990*	-0.231	0.736	0.445
4466-24-4	20534	0.006	0.277	-0.887*	-0.044
57-10-3	985	0.680**	0.680**	0.596*	0.623**
373-49-9	445638	0.568*	0.568*	0.639**	0.708**
544-63-8	11005	0.697**	0.697**	0.499*	0.580*
1002-84-2	13849	0.558*	0.558*	0.310	0.370
6217-54-5	445580	0.681**	0.681**	0.364	0.410
57-11-4	5281	0.709	0.709	-0.854	0.986*
5409-55-2	76420	0.558*	0.426	0.577*	0.509
10396-80-2	146102	0.643**	0.438	0.350	0.418
2437-62-9	270491	0.721**	0.559*	0.316	0.339
52978-85-5	579763	0.483	0.364	0.502*	0.499*
93-55-0	7148	0.600	0.368	0.958**	0.855
1000155-88-8	556457	-0.868	1.000**	-0.270	0.459
122-78-1	998	0.580*	-0.129	0.505	0.387
56829-23-3	5365665	0.239	-0.630*	0.641*	0.452
124-19-6	31289	0.689	-0.996	0.992	1.000**
931-96-4	565407	0.397	-0.262	0.647*	0.504
85896-31-7	522841	0.957*	0.609	-0.576	-0.319
30361-28-5	5283329	0.556	0.940**	0.515	0.437
131-11-3	8554	0.617*	0.469	0.736**	0.689**
2716-53-2	5364833	0.630	0.843**	0.559	0.616
1499-53-2	226558	0.721	0.980	1.000*	0.936
10236-16-5	637195	0.685**	0.293	0.553*	0.523*
628-97-7	12366	0.542*	0.291	0.436	0.375
5487-50-3	554151	0.927*	0.484	0.571	0.622
18240-10-3	86682	-0.152	-0.812*	0.397	-0.044
22117-09-5	5367371	0.942**	0.451	0.699*	0.776*
* 655-48-1	95447	-0.270	-0.253	-0.999*	-0.987

593-45-3	11635	0.532*	0.301	0.079	0.079
629-99-2	12406	0.761**	0.427	0.324	0.395
55282-12-7	292285	-0.126	-0.798**	0.539	0.388
629-92-5	12401	0.559	0.579	0.771*	0.679
7390-81-0	23872	0.783*	0.558	0.758*	0.704
18435-45-5	29075	0.121	0.598*	0.027	0.308
33156-93-3	6440990	0.607*	0.532*	0.464	0.556*
1000150-40-3	5367478	0.620*	0.221	0.359	0.377
56134-03-3	5352709	0.998*	0.999*	0.176	0.053
631-61-8	517165	0.736	0.208	0.882*	0.925**
75-50-3	1146	-0.079	-0.022	0.831*	-0.647
296245-21-1	581431	0.459	0.144	0.635*	0.719**
1000303-08-5	6422106	-0.623	0.433	-0.999*	-0.995

Imported fishmeal

CAS	Compounds CID	Correlation coefficient and significance			
		AV	pH value	VBN	Mold counts
91-20-3	931	0.073	0.594*	0.573*	0.376
93008-87-8	617681	0.349	0.897**	0.753**	0.688**
91-57-6	7055	0.379	0.655*	0.580*	0.624*
65539-79-9	5374980	0.713*	0.191	-0.240	0.187
500-66-3	10377	0.200	0.815**	0.905**	0.339
3208-16-0	18554	-0.673*	-0.036	0.122	-0.497
84679-58-3	614550	0.303	-0.022	0.023	-0.813*
151-10-0	9025	0.757	0.780	0.758	0.865*
1008-88-4	13886	0.653	0.955**	0.958**	0.585
52823-95-7	301207	0.894	0.921	-0.982	0.998*
57-10-3	985	0.201	0.819**	0.730**	0.488
544-63-8	11005	0.373	0.857**	0.808**	0.517*
112-80-1	445639	-0.068	0.632*	0.583*	0.309
1002-84-2	13849	0.294	0.533*	0.494	0.222
373-49-9	445638	0.214	0.832*	0.830*	0.568
100-52-7	240	-0.761**	-0.348	-0.086	-0.639**
638-66-4	12533	0.277	0.871**	0.808**	0.487
2566-90-7	6421262	0.244	0.900**	0.582*	0.536*
1000131-33-2	5363633	0.626	0.993**	0.969**	0.580
2716-53-2	5364833	0.180	0.953*	0.985*	0.405
1000222-86-6	9602988	0.344	0.547*	0.508	0.526
82304-66-3	545303	0.378	0.922**	0.888**	0.683*
29204-24-8	76099200	0.278	0.832*	0.826*	0.716
629-78-7	12398	0.663**	0.616*	0.374	0.486
1000190-92-9	601922	0.447	0.385	-0.511	0.756*
55000-52-7	41282	0.298	-0.483	-0.820*	-0.507
7390-81-0	23872	0.406	0.740**	0.678*	0.521
56554-77-9	557439	0.333	0.932**	0.820**	0.333

137235-42-8	595401	-0.997*	-0.368	0.868	-0.542
18435-45-5	29075	0.959	0.808	-1.000*	0.691
54060-30-9	104682	-0.620	-0.731*	0.419	-0.364

Note:** extremely significant difference, $P < 0.01$; * significant difference，$0.01 < P < 0.05$.
